# Sensing Lipids with Mincle: Structure and Function

**DOI:** 10.3389/fimmu.2017.01662

**Published:** 2017-11-27

**Authors:** Spencer J. Williams

**Affiliations:** ^1^School of Chemistry and Bio21 Molecular Science and Biotechnology Institute, University of Melbourne, Parkville, VIC, Australia

**Keywords:** Mincle, adjuvant, structure–activity relationship, glycolipids, C-type lectin receptor, cell-mediated immunity, innate immunity

## Abstract

Mincle is a C-type lectin receptor that has emerged as an important player in innate immunity through its capacity to recognize a wide range of lipidic species derived from damaged/altered self and foreign microorganisms. Self-ligands include sterols (e.g., cholesterol), and β-glucosylceramides, and the protein SAP130, which is released upon cell death. Foreign lipids comprise those from both microbial pathogens and commensals and include glycerol, glucose and trehalose mycolates, and glycosyl diglycerides. A large effort has focused on structural variation of these ligands to illuminate the structure–activity relationships required for the agonism of signaling though Mincle and has helped identify key differences in ligand recognition between human and rodent Mincle. These studies in turn have helped identify new Mincle ligands, further broadening our understanding of the diversity of organisms and lipidic species recognized by Mincle. Finally, progress toward the development of Mincle agonists as vaccine adjuvants providing humoral and cell-mediated immunity with reduced toxicity is discussed.

C-type lectin receptors (CLRs) comprise a large group of soluble and transmembrane receptors that possess a carbohydrate recognition domain (CRD) or a homologous domain (1). Transmembrane CLRs can function as pattern recognition receptors, enabling the recognition and internalization of a pathogen, its degradation, and subsequently presentation of component antigens, thereby providing innate immune protection and initiating adaptive immunity (2). Mincle is a transmembrane CLR that provides the capacity to recognize a broad range of self- and foreign molecules, as a part of innate immune sensing (Figure 1A) (3–6). Mincle has risen to prominence based on its identification as the key receptor involved in recognition of cord factor and trehalose dibehenate (TDB), trehalose-based lipids with powerful immune modulating properties (7–9). Cord factor is a component of complete Freund’s adjuvant, an emulsion of inactivated and dried mycobacterial cells, and in purified form, can elicit the formation of focal collections of mononuclear phagocyte cells termed granulomas, as well as other serious side effects that have led to it no longer being used for human vaccination (10, 11). TDB is a synthetic analog of cord factor that is the focus of intense interest as an adjuvant for vaccination, particularly when co-formulated with the quaternary amine dimethyldioctadecylammonium (DDA) (12, 13).

**Figure 1:**
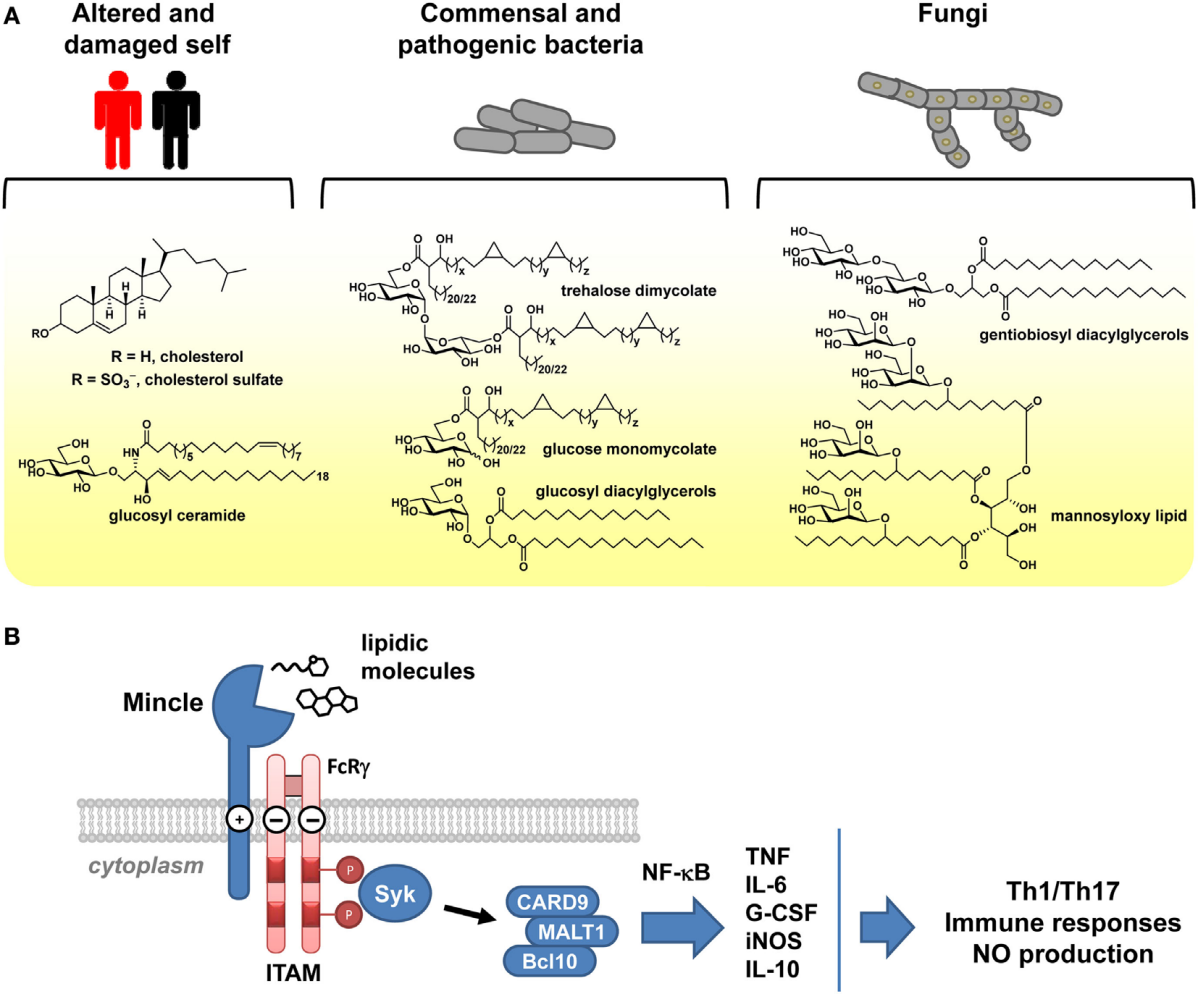
Signaling by Mincle. **(A)** Examples of self and microbial lipidic species that signal through Mincle. **(B)** Signaling through Mincle involves an immunoreceptor tyrosine-based activation motif (ITAM) adaptor and leads to production of cytokines and chemokines, stimulating T-cell and immune responses.

Mincle is a type II transmembrane protein with a short cytoplasmic tail (14) and is expressed on various immune cells including dendritic cells, macrophages, and neutrophils. The extracellular domain binds ligands, causing signal transduction (Figure 1B). Ligand binding occurs at distinct sites, including the CRD and cholesterol and protein interaction sites. Signaling occurs through the immunoreceptor tyrosine-based activation motif-containing Fc receptor γ-chain molecule (15). This process initiates a signaling pathway involving spleen tyrosine kinase (Syk), Card9-Bcl10-MALT1 (16), and finally nuclear factor kappa light-chain enhancer of activated B cells (NF-κB) (2), a transcription factor that promotes the expression of cytokines including tumor necrosis factor (TNF), interleukin-6 (IL-6), granulocyte colony-stimulating factor (G-CSF), and interleukin-11 (IL-10), chemokines including macrophage inflammatory protein 2 (MIP-2 or CXCL2) and KC (CXCL1), as well as stimulating expression of inducible nitric oxide synthase (15, 16). The majority of these responses can be considered as pro-inflammatory; however, production of IL-10 is anti-inflammatory and can lead to down-regulation of IL-12p40 production and interference with pro-inflammatory cytokine secretion (17).

Cytokines produced upon agonism of Mincle signaling orient effector T helper (T_H_) cell development into T_H_ subtypes, although the effects are complex and dependent on the nature of the host and pathogen (18). In mice, a protective T_H_1 cell-mediated immunity is induced, and effects on T_H_17 responses depend on the nature of the pathogen, with cord factor inducing a T_H_17 response. In humans, effects are more complicated. When used as an adjuvant, TDB/DDA induces a T_H_1 response (19). By contrast, assorted fungi such as *Fonsecaea* spp. escape T_H_1-oriented immunity and instead induce a T_H_2 cell-mediated immunity in an NF-κB-independent manner (20); cross-talk with Toll-like receptors can restore T_H_1 immunity (21). Placed in context, these results are important as different T_H_ cell subsets have specific functions in adaptive immunity. T_H_1 and T_H_2 cells provide host immunity against intracellular and extracellular pathogens, respectively, particularly bacteria and protozoa, whereas T_H_17 cells are pro-inflammatory T_H_ cells that are defined by the production of interleukin-17 (IL-17) and play a role in adaptive immunity at mucosal surfaces, especially against fungal pathogens (18).

Aside from the sole exception of the cell death-associated Sin3A-associated protein 130 (SAP130, *vide infra*), signaling through Mincle appears to be limited to water-insoluble glycolipids, with most *in vitro* cell-based studies using plate-bound or crystalline forms of the ligands (7, 22). It seems likely that effective signaling by lipidic species requires multimerization of Mincle at the cell surface and may mimic the presentation of glycolipids on the surface of mycobacterial and other microbial cells and in lipid vesicles (22). This phenomenon appears to be intimately connected with the ability of TDB and TDM as water-in-oil emulsions, or liposomal formulations with DDA, to act as adjuvants.

Mincle is a member of the large family of CLRs that enable recognition of a wide range of self- and foreign ligands (2, 18, 23). Among the CLRs, Mincle is unique in its ability to recognize defined, low molecular weight species and especially glycolipids. As glycolipids are essentially ubiquitous species, there is large scope for Mincle to recognize such species from a wide range of organisms. Our knowledge of the repertoire of lipids that can agonize signaling through Mincle continues to grow, providing growing insight into structure–activity relationships. In addition, a growing range of synthetic lipids have been prepared and studied as agonists of Mincle signaling, further enriching our understanding of the structural features necessary for interaction with this receptor. Collectively, these data show that a remarkable breadth of lipidic species can signal through Mincle (4), suggesting that this receptor has a primitive-like capacity to recognize lipidic species that parallels the Toll-like receptors (24).

## Sensing of Damaged and Altered Self

An important role for Mincle in sterile (non-infected) inflammation has been identified through a range of effector molecules. An early report showed that Mincle is involved in the damaged cell response through recognition of SAP130 (15). Normally, this protein is sequestered within the cell but, upon cellular death, can be released. Binding to SAP130 was shown to occur outside the carbohydrate-binding region of the CRD.

**Figure 2:**
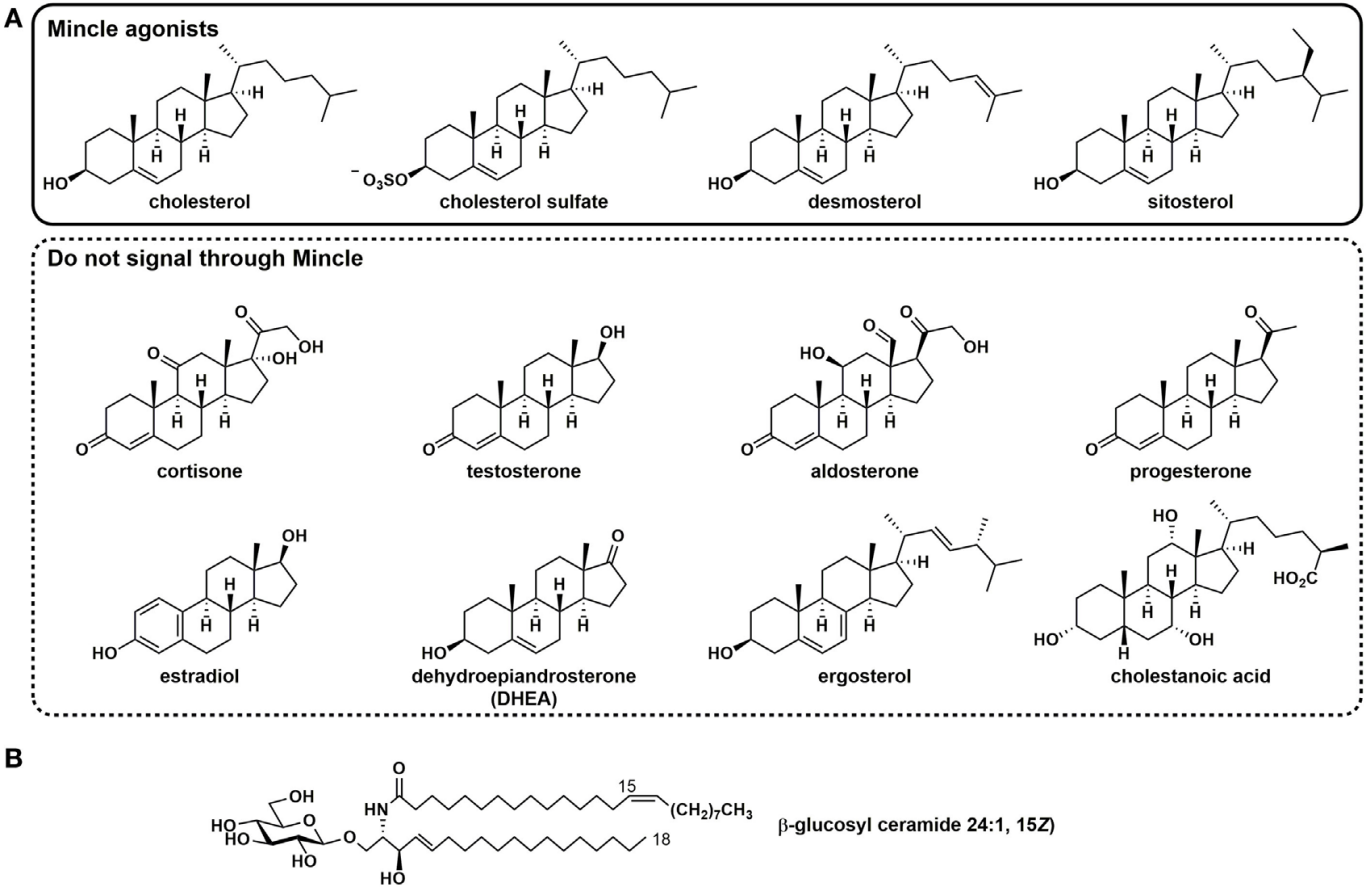
Self-derived lipidic species that signal through human Mincle. **(A)** Sterols that signal through Mincle and assorted steroids that do not signal through Mincle. **(B)** The endogenous glycosyl sphingolipid and β-glucosyl ceramide.

More recently, several self-derived lipidic species have been discovered that signal through Mincle, which cause sterile inflammation (22, 25). Cholesterol crystals, which are present within atherosclerotic plaques during hypercholesterolemia, and within cholesterol granulomas, promote signaling through human Mincle (Figure 2) (22). Analysis of a range of cholesterol esters revealed that only free cholesterol (as either plate-bound or crystalline forms) can signal through human Mincle and that other endogenous steroids such as cortisone, progesterone, estradiol, testosterone, aldosterone, and dehydroepiandrosterone, cannot. Other sterols that can signal through human Mincle include the plant sterol sitosterol and the cholesterol intermediate desmosterol, but the yeast sterol ergosterol and the bile acid cholestanoic acid do not. These results suggest that a hydroxyl residue at C3 and an alkyl chain at C17 appear to be minimally, by not exclusively required for recognition by human Mincle. Recognition of cholesterol is limited to human Mincle; rat and mouse Mincle cannot sense cholesterol. Binding to human Mincle occurs through the cholesterol recognition/interaction amino acid consensus motif L^127^SYKKPKMR^135^, a sequence that is absent in mouse and rat Mincle. The R135L mutant of hMincle lost the ability to recognize crystalline cholesterol but maintained the ability to recognize TDM, suggesting that cholesterol recognition occurs at a site not identical to that of TDM. Only aggregate forms of cholesterol can induce signaling- soluble or membrane-associated forms are inactive- and it was suggested that signaling may be accompanied by multimerization of Mincle.

Cholesterol sulfate, which is present at high levels within the epidermal layer of the skin, has been identified to signal through Mincle and a role for Mincle in allergic skin inflammation has been established (25). Direct binding of cholesterol sulfate to immobilized human Mincle was demonstrated by surface plasmon resonance. Subcutaneous injection of cholesterol sulfate into mice resulted in a Mincle-dependent induction of a severe local inflammatory response with infiltration of neutrophils, monocytes, and eosinophils.

Fractionation of damaged cells led to the discovery that β-glucosylceramide (β-GlcCer), an important host glycosphingolipid, can signal through human and mouse Mincle (26). A range of species including those bearing homologous saturated fatty acyl (C_16:0_,C_18:0_,C_20:0_,C_22:0,C24:0_) and one unsaturated (C_24:1_) variants were detected. Aside from the release of β-GlcCer from cellular damage, this glycosphingolipid is important as it accumulates in Gaucher’s disease, an inherited genetic defect in β-glucosylceramidase (GBA1) that is characterized by systemic inflammation. Mice in which bone marrow dendritic cells (BMDCs) were GBA1-deficient leading to the accumulation of β-GlcCer exhibited enhanced inflammatory responses suggesting that the inflammation-based pathologies of Gaucher’s disease arise from Mincle-mediated processes; this was supported by the observation that no such augmentation was observed in GBA1^−/−^ × Mincle^−/−^ BMDCs. Notably, administration of GlcCer as an oil-in-water emulsion did not induce granuloma formation, suggesting that it may represent a safer compound for therapeutic applications than TDM.

## Sensing of Microbial Lipids from Pathogens and Commensals

### Mycolic Acid- and Corynomycolic Acid-Based Glycolipids

Natural mycolic acids are complex α-alkyl-β-hydroxy fatty acids with the specific structure dependent on the source organism. For example, *Mycobacterium tuberculosis* mycolic acids are C_60–90_ species that include a range of functional groups such as *cis*-alkenes and *cis*- and *trans*-cyclopropanes (CP), ketones, esters, and methoxy groups within the mero chain (27, 28). The equivalent species from corynebacteria are termed corynomycolic acids and are usually simpler, being C_22–38_ in length and containing only saturated and unsaturated species (29, 30).

**Figure 3:**
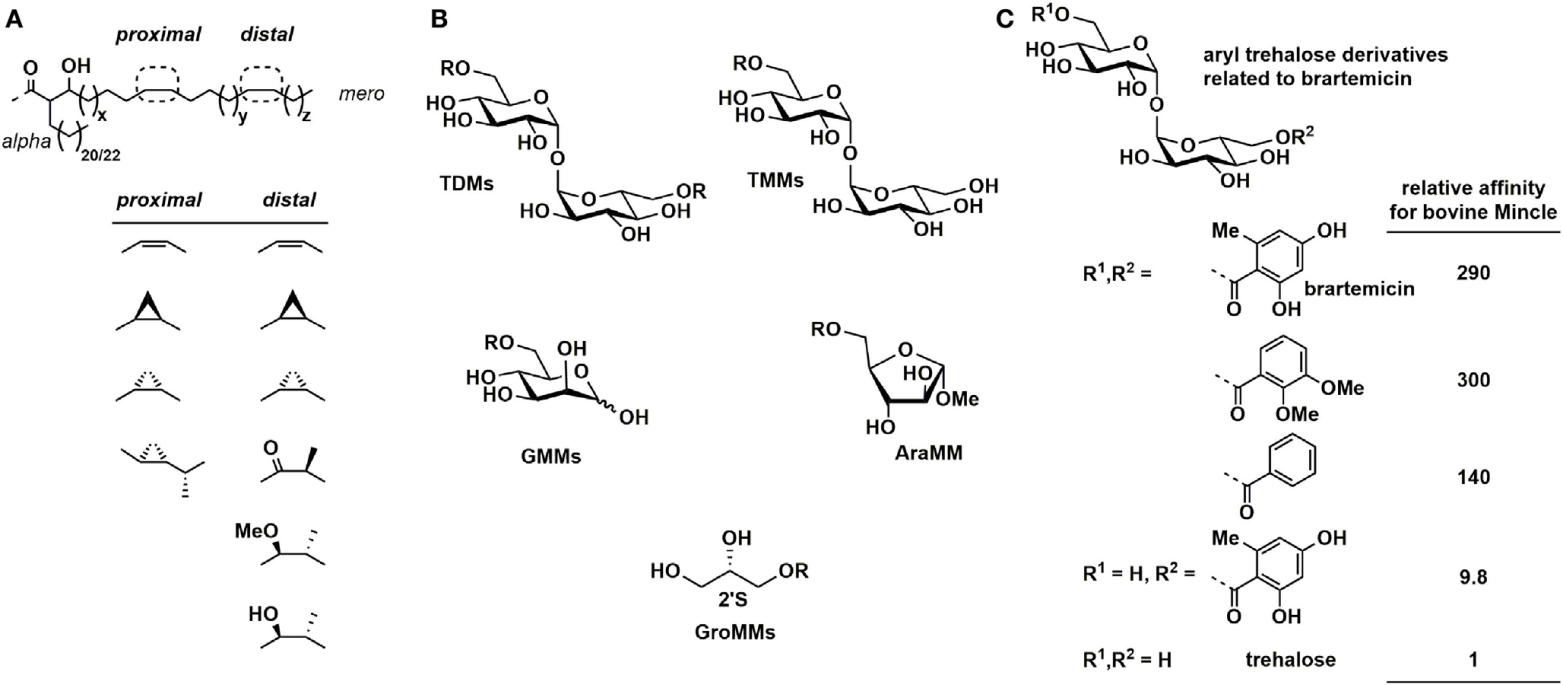
**(A)** Chemically synthesized mycolic acid-based glycolipids: general structures of mycolic acids. **(B)** Mycolic acid-based glycolipid classes. **(C)** Relative affinities of brartemicin and analogs for binding to bovine Mincle.

Baird and co-workers synthesized a large range of homogeneous mycolic acids with structures as shown in Figure 3A (31). These have been elaborated to a range of homogeneous trehalose mono- and dimycolates, glucose monomycolates, and methyl arabinofuranoside monomycolates that bear authentic mycolic acids (Figure 3B), which were examined for their ability to stimulate the production of TNF-α and IL-6 in BMDCs from C57BL/6 mice. Generally, the order of potency was trehalose dimycolates > trehalose monomycolates > glucose monomycolates > arabinoside monomycolates. Most mycolic acid structures induced levels of cytokines similar to natural TDM from *M. tuberculosis*, and greater than that of TDB, except for a *bis*-dialkene TDM that was as potent as TDB. Concerning the lipid fine structure, TDM bearing a *trans*-CP-mero chain was more inflammatory than the equivalent *cis*-CP-mero-chain, while for the oxygenated mycolates, *cis*-isomers were more inflammatory than *trans*-isomers. Among the series of *cis*-isomers, *cis*-methoxy-TDM induced higher TNF-α levels compared to *cis*-alpha TDM or *cis*-keto TDM. These results provide a framework understanding of altered inflammatory activities of TDMs isolated from a range of *M. tuberculosis* mutants that vary in lacking *trans*-cyclopropanation [Δ*cmaA2* mutant (32)], oxygenated MA classes [Mtb Δ*mmaA4* (33)], and α-mycolate cyclopropanation [Mtb Δ*pcaA* mutant (34)], which display hyper- and hypoinflammatory responses.

**Figure 4:**
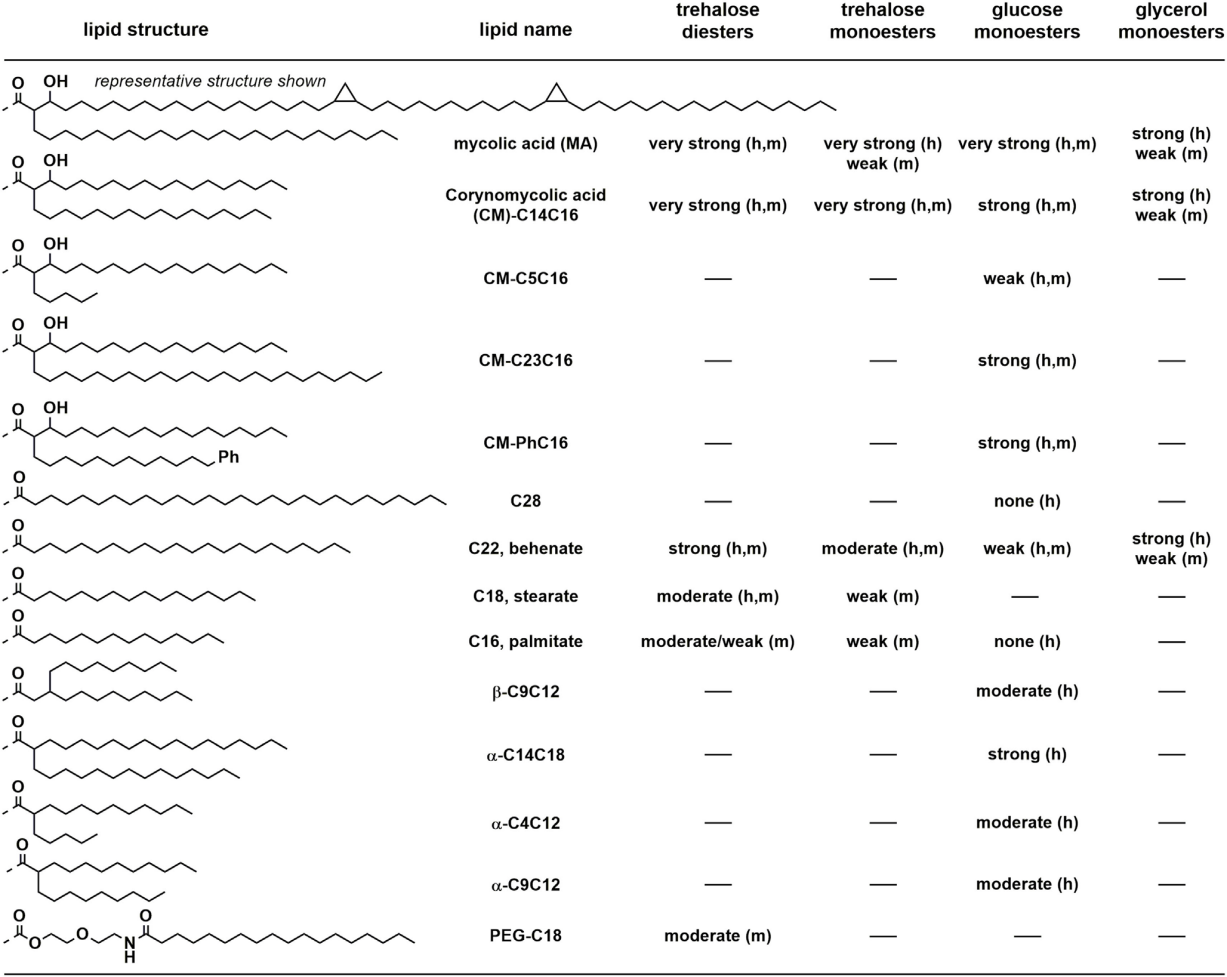
Qualitative summary of structure–activity relationships for human (h) or mouse (m) Mincle-dependent signaling or cellular activation, by trehalose, glucose, and glycerol-based glycolipids. Very strong, strong, moderate, weak, or none refer to signaling through Mincle using reporter cell lines or by production of cytokines and nitric oxide by primary cell lines (usually bone marrow dendritic cells). “–” means not studied.

Lang and co-workers showed that various corynebacteria, including *Corynebacterium diptheriae, C. ulcerans*, and *C. glutamicum*, could bind a murine Mincle-Fc fusion protein (35). Moreover, these bacteria could induce production of the cytokine G-CSF and nitrites by macrophages in a manner analogous to TDB in a Mincle-dependent manner. While the precise glycolipids responsible for these effects were not unequivocally identified, it is likely that they comprise corynomycolate esters of trehalose or glucose. Consistent with this conclusion, a *C. diptheriae* strain (DSM43989), which lacks the ability to produce cell wall mycolates including trehalose dicorynomycolate (TDCM) and trehalose monocorynomycolate (TMCM), or its glycolipid extracts, failed to bind the murine Mincle-Fc fusion and did not active macrophages (Figure 4). Williams and co-workers showed that TMCM, TDCM, and glucose monocorynomycolate (GMCM) derived from a synthetic C_32_-corynomycolic acid representative of *C. glutamicum* could stimulate signaling in a reporter cell expressing human or murine Mincle, at levels similar to that of TDM from *M. smegmatis* (36).

### Simplified Trehalose-Based Glycolipids

Prior to the discovery that cord factor can signal through Mincle, intense effort was applied to the development of structurally simpler and safer immunomodulators than whole heat-killed mycobacterial cells (11, 37). These studies demonstrated that adjuvant effects could be obtained with TDM, a molecule that had previously been identified as capable of inducing formation of granulomas that characterize lung infection with *M. tuberculosis*, and further studies showed that simplified cord factor analogs possessed useful adjuvant capabilities, often with better safety, particularly in the context of granuloma formation (10). As a part of efforts to develop less toxic analogs of TDM, TDB was invented (38). TDB is a purely synthetic simple analog of TDM that has been widely employed in vaccination studies (39). In independent studies, TDM and TDB were shown to signal through Mincle (7, 9).

Stocker and co-workers studied the ability of trehalose diesters of simple straight chain fatty acyl lipids to activate murine bone marrow-derived macrophages (BMMs) (40). Short-chain saturated (C_4_, C_7_, and C_10_) derivatives did not stimulate NO production, while C_18_, C_20_, C_22_, and C_26_ were able to produce NO and the cytokines IL-6 and IL-1b, with a maximum activity for C_22_ (TDB). Trehalose C_22_ and C_26_ monoesters were also shown to activate BMMs but with lesser potency relative to the equivalent diesters; production of NO but not IL-6 was dependent on prestimulation with IFN-γ. Generally in these assays, the C_22_ monoester was more potent than the C_26_ monoester (41).

Huber and co-workers studied a series of trehalose mono- and diesters bearing C_14_ to C_22_ fatty acyl chains for their ability to activate BMMs and BMDCs (42). In terms of production of nitrite and G-CSF, the diesters were superior to the monoesters. Dose–response curves for trehalose monoesters and diesters confirmed the differences in potency in BMMs; an approximately 100-fold higher concentration of the monoester was required for robust production of nitrites yet still failed to induce G-CSF. Similar differences were seen in direct binding assays of a mouse Mincle-Fc to plate-coated mono- and diesters. These authors also studied a trehalose distearate (TDS) derivative that included a short PEG-spacer (PEG-C18). This compound exhibited significantly reduced potency for macrophage activation, relative to the distearate, yet still maintained activity greater than that of the stearate monoester.

Mycobacteria produce a wide range of other trehalose-based glycolipids. Decout et al. studied a range of glycolipids extracted from *M. tuberculosis* H37Rv (43). Trehalose derivatives that are acylated at the 2 and 3 positions such as diacyltrehalose and diacylglycolipid Ac_2_GL (which is derived from Ac_2_SGL by mild acid treatment) and at the 2, 3, and 6′ positions (triacyltrehalose) with various combinations of straight chain and methyl-branched fatty acids provide at best modest signaling through Mincle. Notably, the 2′-sulfated form of Ac_2_GL, Ac_2_SGL, does not signal through Mincle nor do a range of other trehalose-based glycolipids that lack a free 2-hydroxyl group. Collectively, these results reveal that the presence of a free trehalose 2-hydroxy group is critical for trehalose glycolipid signaling through Mincle.

Binding studies of trehalose analogs to the CRD of Mincle have by matters of practicality been limited to soluble derivatives, which typically do not signal through Mincle. Nonetheless, such binding data are important as it provides direct information on the affinity of the protein for the monomeric soluble glycolipid. Competition studies of binding of various soluble carbohydrate species to bovine Mincle have revealed key features required for tight binding to the CRD. Trehalose binds some 36-fold more tightly than methyl α-d-glucopyranoside, highlighting the importance of two sugar-binding subsites (44). Increasing the chain length of C_2_–C_6_ trehalose diesters resulted in a monotonic increase in enhancement of binding relative to trehalose of up to 250-fold (44, 45). Activity for a similar series of trehalose monoesters from C_4_ to C_12_ increased monotonically up to 530-fold greater than trehalose. Surface plasmon resonance was used to probe binding of immobilized human Mincle CRD to soluble C_8_, C_10_, and C_12_ trehalose monoesters and showed monotonically increasing binding affinities (46). A plot of the binding affinities for the number of carbons per substituent for mono-acyl and di-acyl trehaloses versus log *K_i_* was linear for a series of linear, iso-branched, and aromatic groups (45).

Drickamer and co-workers reported the synthesis of a glycan array containing the major carbohydrate structures present in the cell wall of *M. tuberculosis* and other mycobacteria (47). This array was probed with fluorescently labeled bovine Mincle, or alternatively by a secondary antibody. Bovine Mincle interacted with high selectivity only with trehalose-based structures, including immobilized trehalose monomycolate. Additionally, binding was seen for more complex glycan structures including a β-glucosyl-1,6-trehalose that comprises the core of several *M. smegmatis* lipooligosaccharides, and trehalose derivatives modified at the 4-position with additional β-glucosyl residues that comprise the glycan core of surface lipooligosaccharides from the opportunistic pathogen *M. kansasii*.

Brartemicin is a trehalose-based natural product from *Nonomuraea* sp. that has been reported to inhibit matrigel invasion of cancer cells (Figure 3C) (48). Brartemicin has been studied as a soluble analog of cord factor for binding to soluble bovine Mincle (49). In a competition binding assay, brartemicin and related analogs bound 300-fold tighter to Mincle than trehalose and trehalose dibenzoate bound around 140-fold more tightly. Interestingly, a monoester derivative bearing only one of the 2,4-dihydroxy-6-methylbenzoyl groups of brartemicin bound only 10-fold more tightly than trehalose, and *epi*-brartemicin, with an αβ-trehalose core, bound 3-fold more tightly than trehalose (i.e., 1/10th the affinity of brartemicin). It has not been reported whether brartemicin can signal through Mincle.

### Glucose- and Glycerol-Based Glycolipids

The mycobacterial metabolite glycerol monomycolate (GroMM) has been shown to signal through human Mincle but not mouse Mincle (Figure 4) (50). Glycerol monobehenate (GroMB) also selectively signals through human Mincle but somewhat less potently than GroMM (50). Glycerol monocorynomyclate (GroMCM), a shorter-chain, C_32_-corynomycolate analog, also signals through human Mincle selectively over mouse Mincle, and with similar potency to GroMM (36). Glycerol monoesters can exist in two stereoisomeric forms at the glycerol. In the case of GroMCM, the majority of the activity resides in the 2′*S*-isomer (36). Baird and co-workers have prepared a series of 2′*R*- and 2′*S*-GroMMs bearing authentic α-, keto-, and methoxy-mycolic acids (51). Consistent with the results for the GroMCMs, the 2′*R*-isomers did not show any significant effects in the stimulation of cytokines in BMDCs.

An early report noted that trehalose dimycolate, upon treatment with porcine trehalase, lost the ability to signal through Mincle (7). Although originally interpreted as indicating that GMM cannot signal through Mincle, it is now clear that this reduction of activity is not as a result of formation of GMM and may be as a result of contaminating esterase activity. Decout et al. reported that GMM isolated from *M. tuberculosis* H37Rv was a powerful agonist of human and mouse Mincle reporter strains, with potency greater than that of TDM (43). Synthetic GMMs bearing authentic homogeneous mycolic acids were synthesized by Baird and co-workers and were able to activate BMDCs (31). A range of glucose monomycolate analogs have been synthesized. Detailed examination of the lipid structure while maintaining the α-alkyl-β-hydroxy motif has revealed that GMCM is a strong Mincle agonist and that activity is maintained upon increasing the length of the α-chain or incorporation of an aryl group into this chain (52). In contrast, shortening of the α-chain to a pentyl group resulted in the loss of signaling through Mincle (52).

More extensive analysis of the effect of structure on signaling through Mincle for GMM analogs reveals a complex dependence on structure. In contrast to the potent signaling seen for TDB, TMB, and GroMB, glucose monobehenate is only a very weak activator of human and mouse Mincle reporter cells (36, 43, 52). A β-branched C9C12 derivative signaled only weakly through human or mouse Mincle (52). Short, α-branched lipids were also able to signal through Mincle but short- to medium-chain derivatives were only moderate agonists (43). A longer chain α-C14C18 analog provided strong signaling through Mincle approaching the potency of GMCM, and the mannose analog also provided robust, albeit slightly weaker signaling through Mincle. This last result is interesting in the context that a mannose monomycolate derived from *Rhodococcus ruber* (*Nocardia rubra*) with an intermediate C_45_ mycolic acid did not induce granulomas in mice when delivered as a water-in-oil-in-water emulsion (53).

### Overview of Glycolipid Recognition by Mincle

Several X-ray structures are available for ligands bound to Mincle from bovine (44, 45) or human (46) sources. These structures reveal binding of trehalose or simple trehalose derivatives in the CRD of Mincle. The CRD contains two carbohydrate-binding sites, with one appearing to act as a “primary” binding site and involving interactions of the O2 and O3 of a glucose residue with the Ca^2+^ ion; the secondary sugar-binding site does not interact with the Ca^2+^ ion. A lipophilic groove extends away from the 6 position of the glucose residue in the primary binding site that modeling studies suggest can accommodate two lipid chains (45). Taken together, structure–function relationship studies, crystallography, and molecular modeling data suggest that effective signaling through Mincle by glycolipids can involve interactions with one or both sugar-binding subsites and with one or two alkyl chains present on the same or different fatty acids and that the 2-hydroxy group must not be modified. Decout et al. have argued that at least three of these four binding sites should be occupied for effective signaling to occur (43). The fact that mono-acyl trehaloses appear to be less effective Mincle signaling agonists than the branched glycolipid GlcC14C18 suggests that the interactions with the secondary sugar-binding site are less important than those with a second alkyl chain in the second hydrophobic groove.

### Glycosyl Glycerolipids

**Figure 5:**
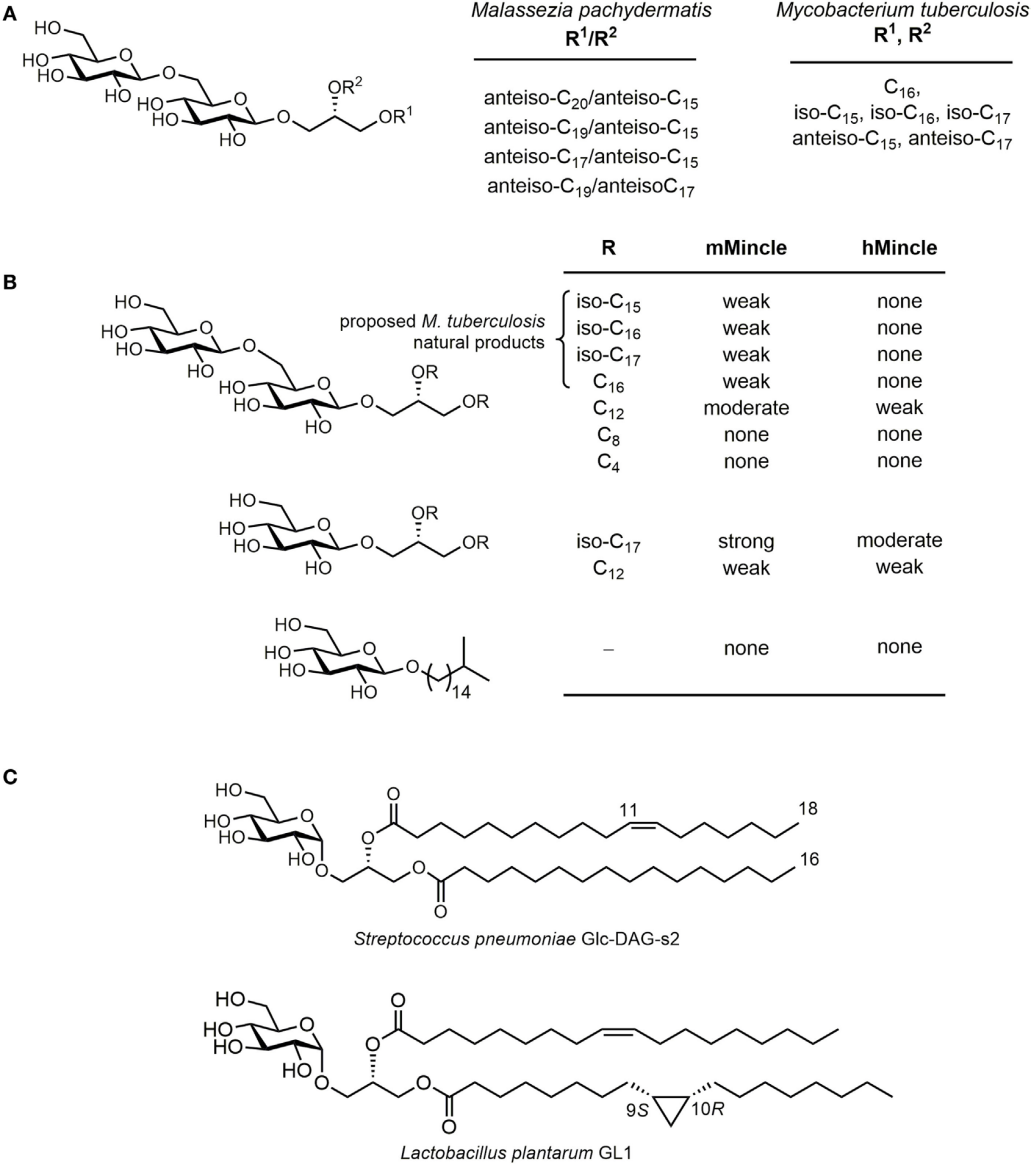
Glycosyl diacylglycerols are a class of microbe-associated molecular patterns that signal through Mincle. **(A)** Structures of β-gentiobiosides isolated from *Malassezia pachydermatis* that weakly signal through mouse Mincle and related compounds from *Mycobacterium tuberculosis* H37Ra. **(B)** Structure activity relationships for signaling through mouse and human Mincle for β-glycosyl diglycerides using reporter cell assay. **(C)** Structures of α-glucosyl diglycerides from pathogenic and commensal bacteria that signal through Mincle.

Yamasaki et al. screened 50 species of pathogenic fungi for their ability to signal through Mincle using a murine Mincle-GFP reporter strain and discovered signaling by a range of *Malassezia* spp., including *M. pachydermatis* and *M. dermatis* (54). In normal skin, *Malassezia* spp. are commensals; however, in atopic/eczema and psoriasis, these fungi can elicit inflammatory responses in skin lesions and can cause diseases such as tinea versicolor, atopic dermatitis, and lethal sepsis. Fractionation of a lipid extract from *M. pachydermatis* led to the identification of two classes of Mincle agonists (55). The first comprised a complex mannosyloxystearyl mannitol that possessed a potency similar to that of TDM (Figure 1). Limited degradation studies revealed that the mannosyloxystearic acid fragment was a weak Mincle agonist. The second class of Mincle agonist was a series of β-gentiobiosyl diglycerides (Figure 5A). Four lipoforms were isolated with the following substituent permutations (sn-1/sn-2): anteiso-C_19_/anteiso-C_15_, anteiso-C_17_/anteiso-C_15_, anteiso-C_20_/anteiso-C_15_, and anteiso-C_19_/anteiso-C_17_. All four lipoforms signaled weakly through mouse Mincle to similar degrees but did not signal through human Mincle. The *Malassezia* β-gentiobiosyl diglycerides have structures similar to that of the gentiobiosyl glycolipid anchor of lipoteichoic acids, a major constituent of the cell wall of Gram-positive bacteria; however, lipoteichoic acid does not signal though Mincle.

**Figure 6:**
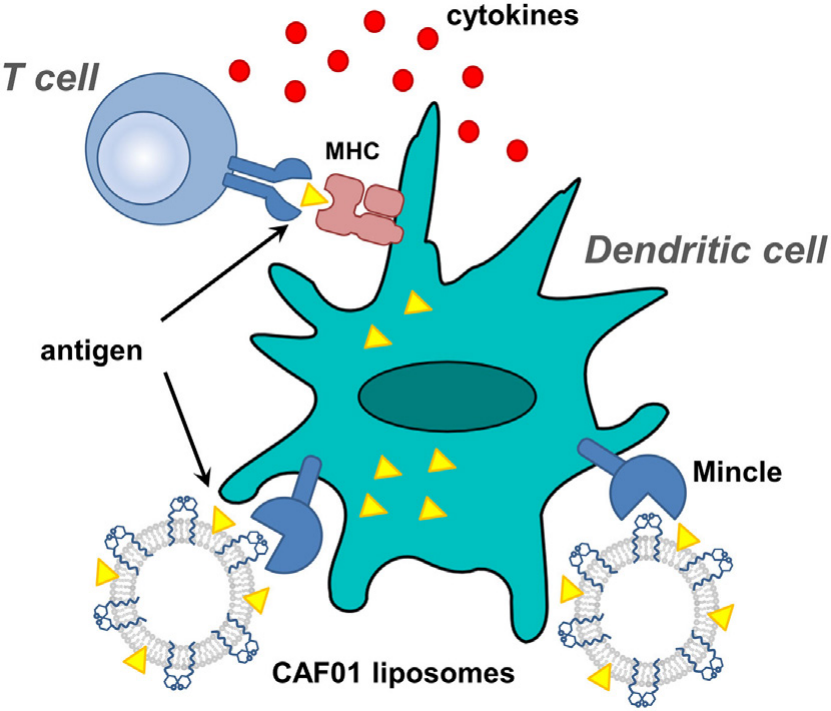
Proposed action of CAF01-adjuvanted antigen vaccination. Liposomes, composed of dimethyldioctylammonium bromide liposomes co-formulated with TDB, are emulsified with antigen and used to stimulate dendritic cells.

A related series of gentiobiosides were isolated by Brennan and co-workers from *M. tuberculosis* H37Ra (56). In order to determine whether these compounds can signal through Mincle, Williams and co-workers synthesized a series of related gentiobiosides representing those isolated, namely four lipoforms bearing C_18_, iso-C_17_, iso-C_18_, and iso-C_19_ fatty acids (Figure 5B) (57). Like the *Malassezia* gentiobiosides, all four lipoforms signaled only weakly through mouse Mincle and did not signal through human Mincle. A range of analogs were synthesized to explore structure–activity relationships. The branched alkyl gentiobioside, the glycerol gentiobioside, and short-chain analogs bearing C_4_ or C_8_ fatty acyl chains did not signal through Mincle. However, an analog bearing two C_12_ chains provided moderate signaling through mouse Mincle and also provided weak signaling through human Mincle. Among the most potent of the analogs examined was a β-glucosyl diglyceride bearing iso-C_17_ fatty acids, which provided moderately strong signaling though mouse Mincle and moderate signaling through human Mincle. This compound was proposed to represent a biosynthetic precursor to the mycobacterial β-gentiobiosides, suggesting that human and mouse Mincle can preferentially recognize β-glucosyl diglycerides rather than their extended derivatives.

α-Glucosyl diacylglycerides from the pathogenic bacterium *Streptococcus pneumoniae* were shown by Yamasaki and co-workers to trigger Mincle reporter cell activation (Figure 5C) (58). These glycolipids are comprised of a range of lipoforms, including a C_16:0_/C_18:1_ species where C_18:1_ is *cis*-vaccenic acid (59, 60). Model synthetic α-GlcDAGs bearing myristic (C_14:0_/C_14:0_) or stearic acid esters (C_18:0_/C_18:0_) could signal through Mincle, with the former more potent than the latter. Like β-gentiobiosyl diglycerides, α-glucosyl diacylglycerides are also lipid anchors of lipoteichoic acid, which do not signal through Mincle. Williams and co-workers synthesized an α-glucosyl diglyceride from the commensal bacterium *Lactobacillus plantarum* (61), which contained a CP fatty acid (dihydrosterculic acid) and oleic acid and demonstrated that this could activate human and mouse Mincle NFAT-GFP reporter cells, with similar potency (62, 63). A range of analogs were prepared that lacked cyclopropanation and/or unsaturation; all signaled though human and mouse Mincle with similar potency.

Collectively, these results suggest that α- and β-glucosyl diacylglycerides comprise a class of microbe-associated molecular patterns that can signal through Mincle.

### Adjuvant Studies

Growing interest in the development of subunit vaccines for infectious diseases and cancer has intensified interest in the development of adjuvants (64, 65). Instead of the entire microbe, subunit vaccines include only the antigens that stimulate the body, in some cases being restricted to the specific antigen that is recognized by T cells or antibodies. Benefits of subunit vaccines include a lower potential for adverse reactions, the ability to be produced using recombinant DNA technology (recombinant subunit vaccines) or by chemical synthesis (molecular subunit vaccines), and the ability to systematically alter their structure (and therefore function) (66). However, subunit vaccines are inherently less immunogenic because they lack a full suite of cellular or viral immunogens and co-administration of an adjuvant is often required to stimulate a potent immune response.

A critical role for an oil delivery agent for trehalose glycolipid immunogens was demonstrated in the earliest studies of the ability of mycobacterial cells to act as adjuvants and immunostimulants. For example, complete Freund’s adjuvant is an emulsion of inactivated and dried mycobacterial cells in various vegetable and mineral oils, which is emulsified with antigen using the surfactant mannide monooleate (11). To identify the bioactive component, Bloch obtained petroleum extracts of corded *M. tuberculosis* H37Rv or *M. bovis* Vallée to obtain a “cord factor” that alone was toxic to mice when injected as a suspension in paraffin oil (67). Subsequently, trehalose diesters have most commonly been studied as oil-in-water emulsions, with variation in the nature of the oil (mineral/paraffin, vegetable, squalene, or squalane), droplet size, and amount of Tween detergent (10). These changes can have large effects on immunogenicity as well as toxicity, with larger droplets often giving more severe reactions such as granulomas (68). Use of oil-in-water emulsions for intraperitoneal or intramuscular injection is believed to act through three different mechanisms: the establishment of a local depot at the site of injection allowing for sustained continuous release of the antigen; provision of a vehicle for transporting antigen through the lymphatic system; and interaction with immune cells such as antigen-presenting and phagocytic cells (11). The long duration of oil-in-water emulsions is striking: subcutaneous injection of squirrel monkeys or rats with radiolabeled hydrocarbon emulsified with mannide monooleate revealed that after 10 months nearly 30% of radiolabeled hydrocarbon was still located at the injection site (69).

Early studies of TDM analogs as immunomodulators have been reviewed (10). TDCM as an oil-in-water emulsion was shown to protect against bacterial challenge and suppress tumor growth. Typically, it was found to be similarly effective as TDM. Early studies with simple trehalose diesters utilized oil-in-water emulsions (using vegetable and mineral oils). TDB was found to be more effective than trehalose dipalmitate in suppressing ascetic tumor growth and protecting against bacterial infection. Typically, TDB was less effective than TMCM in suppressing tumor growth and protecting against bacterial infection; the reversed potency was seen for suppressing ascetic tumor growth in rats.

A breakthrough was made in the development of a two-component adjuvant comprised TDB and DDA, which can confer strong humoral and cell-mediated immune responses (39, 70). Alone, DDA acts as an adjuvant that can be co-formulated with antigens to elicit strong cell-mediated and moderate-to-strong humoral responses (71). DDA forms liposomes, but these are relatively unstable and aggregate. However, upon co-formulation with TDB, the liposomes exhibit greater stability and adjuvanticity, with optimum levels of IFN-γ production at 11% TDB; this formulation is termed CAF01 (70). Evidence that the adjuvancy arises from signaling through Mincle was obtained by showing that Mincle^−/−^ mice could not be adjuvanted to H1 (Ag85B-ESAT-6) subunit vaccination by CAF01 (9). Immunization of mice with H1 in DDA–TDB liposomes induced a strong, specific T_H_1 biased immune response characterized by substantial production of the interferon-γ cytokine and high levels of IgG2b isotype antibodies (39). More extensive studies of the CAF01 adjuvant has characterized it as a unique adjuvant, with low toxicity, and capable of providing a T_H_1/T_H_17 profile that is distinct to other approved (alum, squalene – MF59) and promising (IC31, GLA-SE) adjuvants and is effective in enhancing responses to *M. tuberculosis*, chlamydia, and HIV-derived peptides (12).

Given that no approved human adjuvants are available for induction of cellular immunity, CAF01 has attracted keen interest. A Phase I study was reported that enrolled healthy human volunteers who were vaccinated with the H1 and adjuvanted with CAF01 (19, 72). Two vaccinations elicited strong antigen-specific T-cell responses that persisted after 150 weeks, indicating the induction of a long-lasting memory response. CAF01 was shown to be a safe and tolerable T_H_1-inducing adjuvant for human vaccination studies in where cellular immunity is required.

Other Mincle agonists have been investigated as CAF01-like adjuvants. Decout et al. examined the ability to adjuvant Ag85A immunization in mice (43). The glycolipids TDB, GlcC14C18, and ManC14C18 were formulated in a 1:25 ratio with DDA, which is suboptimal for TDB/DDA. For TDB/DDA, little effect on IL-2, IFN-γ, or IL-17 production was observed relative to DDA alone. Enhanced production of these cytokines was seen for ManC14C18 but without statistical significance. Significant increases in the cytokines were seen for GlcC14C18. DDA/GlcC14C18 provided superior production of Ag85A-specific IgG2b titers, 80-fold greater than for DDA alone, while IgG1 titers were unaffected, a pattern characteristic of T_H_1 response. A 25:1 DDA/GlcC14C18 formulation was as effective as the optimal 10:1 DDA/TDB formulation in the induction of protective immunity to *M. tuberculosis* infection, suggesting enhanced potency of the GlcC14C18 glycolipid.

Huber et al. have studied simple trehalose diesters for the ability to adjuvant *Chlamydia trachomatis* serovar D major outer membrane protein (42). TDS, trehalose monostearate (TMS), TDB, and trehalose PEG-C18 were formulated in an 11% ratio with DDA, which is optimal for TDB (70). Following immunization, TDB and TDS were more effective for the production of IL-17a and IFN-γ from splenocytes than TMS and PEG-C18 and produced higher numbers of antigen-specific IFN-γ^+^ and IL-17a^+^ CD44^+^CD4^+^ T cells. A study that evaluated the ability of TDP, TDS, and TDB to adjuvant H56 (Ag85B-ESAT-6-Rv2660c fusion protein) immunization of mice found all three glycolipids (as an 11% formulation in DDA) elicited comparable T-cell responses (73).

## Conclusion

The discovery that Mincle is the key receptor involved in signaling by TDM and TDB has stimulated growing interest in the discovery of agonists for this receptor. A growing repertoire of natural lipidic species has been identified that belong to three major classes: sterols (e.g., cholesterol); trehalose, glucose, GroMMs, and related acylated species; and glycosyl diglycerides. Molecules from these classes are produced by an assortment of pathogenic and commensal microorganisms, suggesting the involvement of Mincle signalling in a broad range of infectious diseases and in a healthy gut microbiota. Rational design of Mincle agonists based on these structural templates is now possible and has led to the development of simplified structures that achieve similar or improved levels of immune stimulation as for natural cord factor. The dual humoral and cell-mediated immunity induced by CAF01 is supportive for its application as an adjuvant for vaccines directed at the treatment of infectious disease and holds promise for application in cancer immunotherapy, for example, in adjuvanting tumor-associated carbohydrate antigen vaccines. Finally, while attention to date has focused on developing small molecule agonists of Mincle signaling, future efforts should seek to discover small molecule antagonists of Mincle signaling in conditions of sterile inflammation, which could assist in probing the role of Mincle in diseases such as stroke (74), atherosclerosis (22), Gaucher’s disease (26), skin allergies (25), and hepatitis (75).

## Author Contributions

SW takes full responsibility for this article.

## Conflict of Interest Statement

The author declares that the research was conducted in the absence of any commercial or financial relationships that could be construed as a potential conflict of interest.
